# Sequence to Medical Phenotypes: A Framework for Interpretation of Human Whole Genome DNA Sequence Data

**DOI:** 10.1371/journal.pgen.1005496

**Published:** 2015-10-08

**Authors:** Frederick E. Dewey, Megan E. Grove, James R. Priest, Daryl Waggott, Prag Batra, Clint L. Miller, Matthew Wheeler, Amin Zia, Cuiping Pan, Konrad J. Karzcewski, Christina Miyake, Michelle Whirl-Carrillo, Teri E. Klein, Somalee Datta, Russ B. Altman, Michael Snyder, Thomas Quertermous, Euan A. Ashley

**Affiliations:** 1 Stanford Center for Inherited Cardiovascular Disease, Stanford University, Stanford, California, United States of America; 2 Stanford Cardiovascular Institute, Stanford University, Stanford, California, United States of America; 3 Division of Cardiovascular Medicine, Stanford University, Stanford, California, United States of America; 4 Division of Pediatric Cardiology, Stanford University, Stanford, California, United States of America; 5 Stanford Center for Genomics and Personalized Medicine, Stanford University, Stanford, California, United States of America; 6 Department of Genetics, Stanford University, Stanford, California, United States of America; 7 Biomedical Informatics Training Program, Stanford University, Stanford, California, United States of America; Harvard Medical School, UNITED STATES

## Abstract

High throughput sequencing has facilitated a precipitous drop in the cost of genomic sequencing, prompting predictions of a revolution in medicine via genetic personalization of diagnostic and therapeutic strategies. There are significant barriers to realizing this goal that are related to the difficult task of interpreting personal genetic variation. A comprehensive, widely accessible application for interpretation of whole genome sequence data is needed. Here, we present a series of methods for identification of genetic variants and genotypes with clinical associations, phasing genetic data and using Mendelian inheritance for quality control, and providing predictive genetic information about risk for rare disease phenotypes and response to pharmacological therapy in single individuals and father-mother-child trios. We demonstrate application of these methods for disease and drug response prognostication in whole genome sequence data from twelve unrelated adults, and for disease gene discovery in one father-mother-child trio with apparently simplex congenital ventricular arrhythmia. In doing so we identify clinically actionable inherited disease risk and drug response genotypes in pre-symptomatic individuals. We also nominate a new candidate gene in congenital arrhythmia, *ATP2B4*, and provide experimental evidence of a regulatory role for variants discovered using this framework.

## Author Summary

Technological advances have dramatically reduced the cost of sequencing the human genome. Tools for analyzing such data across families including annotation of clinically important variants and aggregation of variants for personalizing drug prescriptions have been developed but few are publically available. Here we describe such tools then demonstrate their application in several distinct data sets. In particular, we use the tools to define the genetic basis of a new congenital arrhythmia syndrome.

## Introduction

Since the completion of the human genome project, technological advances have dramatically increased throughput and decreased the cost of human DNA sequencing[1], facilitating comprehensive interrogation of coding regions of the genome, transcripts, and whole genome sequences. High throughput sequencing has illuminated the underlying genetic basis for rare inherited disease syndromes[2–4], refined our molecular understanding of cancer pathogenesis[5]. provided a fine map of rare genetic variation underlying common disease risk[6–9], and refined clinical diagnosis and medical therapy[10–13].

These initial advances and the continued drop in the cost of high-throughput sequencing have prompted predictions of a new era of medicine personalized to individual genetics. However, downstream interpretation of sequence variation data remains a formidable barrier to full realization of the promise of genomic medicine, whether it be applied for investigating the genetic basis for well-described disease phenotypes in individuals and families or for prognostication of disease risk and drug response[1]. Several applications and data resources exist for predicting the effects of genetic variation on human phenotypes[14–17], but there does not yet exist a comprehensive, widely accessible application for interpretation of whole genome sequence data. We previously developed and applied a methodology for interpretation of genetic and environmental risk in a single participant using a combination of traditional clinical assessment, whole genome sequencing, and integration of genetic and environmental risk factors[18], and extended this framework to familial context[19]. Here we describe an integrated pipeline, Sequence To Medical Phenotypes (STMP) for interpreting high-throughput human DNA sequence data. STMP performs targeted genotyping of variants with known clinical associations, rich functional annotation of discovered variants, and prioritizes genetic variants according to potential impact, mode of inheritance, and phenotypic presentation. For individual genome sequences, STMP provides predictive genetic information regarding risk for inherited disease traits and response to pharmacological therapy. We demonstrate the use of this analytical pipeline for disease and drug response prognostication in pre-symptomatic individuals, and for elucidation of the genetic basis congenital ventricular arrhythmia.

## Methods

### Ethics statement

This study was reviewed and approved by the Research Compliance Office at Stanford University (protocol # 4237, SQL 96726). Informed written consent was obtained from all participants.

### STMP heuristic

**Fig 1:**
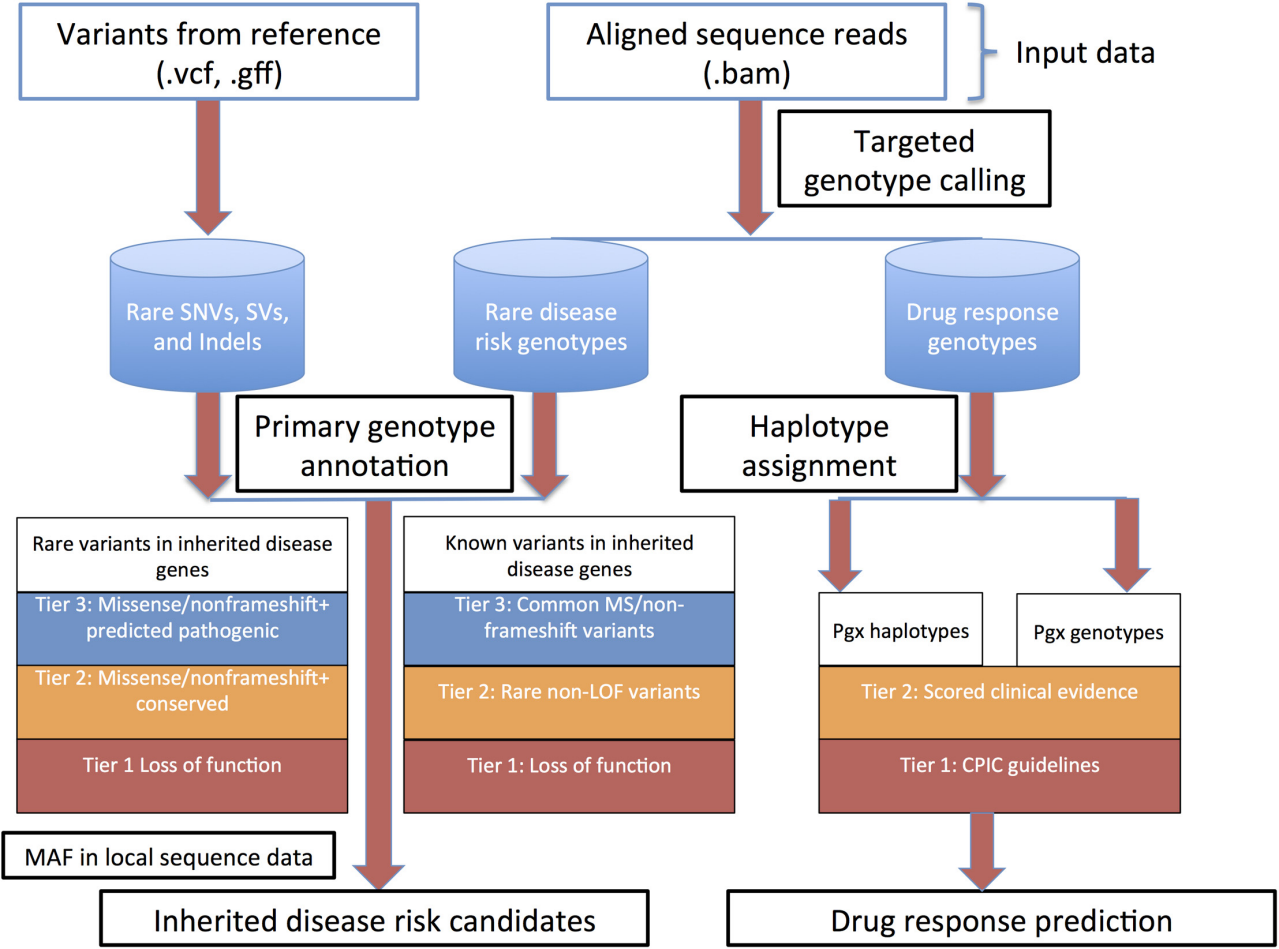
Overall heuristic for variant identification, genotype determination, initial annotation and downstream prioritization. Abbreviations: CPIC, clinical pharmacogenomics implementation consortium; LOF, loss of function; MAF, minor allele frequency; Pgx, pharmacogenomics; SNVs, single nucleotide variants; SVs, structural variants.

Methods for whole genome sequence (WGS) interpretation in the context of disease gene finding in inherited disease syndromes and predictive genetic variant annotation are outlined in heuristic fashion in **Fig 1.**

### Genotyping of clinically relevant variants

High-throughput re-sequencing currently requires a reference genome for sequence assembly and variant identification. The reference genome that is currently used for alignment of human re-sequencing data and variant identification (the NCBI reference genome)[20] is derived from a collection of DNA samples from a small number of anonymous volunteers. However, it represents a very small sampling of human genetic variation. As such, at ~1.6 million genomic positions, the NCBI reference sequence differs from the major allele in each of the three Haplotype Map (HapMap) populations. These minor alleles span ~4,500 variant positions associated with common complex disease and drug response traits[19], including the Factor V Leiden allele associated with hereditary thrombophilia. Comparison of genome sequence data that is homozygous for these alleles to this reference sequence will naturally not produce a variant call. This issue is partially addressed by the use of major allele reference sequences[19]. A more comprehensive approach is to perform targeted genotype calling of all loci considered to be of phenotypic importance. This approach is reference agnostic up to reference base bias associated with short read alignment and mapping. STMP uses input interval call files representing previously reported Mendelian disease associated loci and loci associated with drug response to provide targeted genotype calls, irrespective of reference base, using the GATK Unified Genotyper (for SNVs) and Haplotype Caller (for indels) and their capability to report genotypes and coverage for the “reference calls”. STMP also provides metrics for coverage of loci with known importance to human health and disease, thereby providing confidence that, for example, a given disease-associated allele is indeed not present, rather than just under-sequenced or otherwise not confidently ascertained. As compared with methods that store diploid calls for all reference genomic positions, the STMP approach to genotype interrogation facilitates downstream variant annotation while minimizing storage requirements for genotype data. To facilitate updated genotype identification as new loci of relevance to human health and disease are discovered, binary alignment (BAM) files from the secondary sequence analysis are retained for future use.

### Functional annotation of genetic variants

Rich functional genomic annotation is a prerequisite to sequence interpretation pipelines that aim to provide testable biological hypotheses about the basis for described disease syndromes and for disease risk and drug response prognostication. We extended the annovar framework [21] to provide rich gene-based, functional genomic, regulatory, allele frequency, and phenotypic annotation. This basic annotation pipeline provides 94 annotations for SNVs and indels in VCF format (http://www.1000genomes.org/wiki/Analysis/Variant%20Call%20Format/vcf-variant-call-format-version-41, **S1 Table**) and 39 annotations for SVs in GFF format (https://www.sanger.ac.uk/resources/software/gff/spec.html **, S2 Table**).

STMP can also leverage gene co-expression network topology information to provide quantitative prior expectations about gene-level pathogenicity for contextualizing individual variation data. For example, STMP output may be used to identify genetic variation occurring in genes that are co-expressed with known disease genes, thereby implicating by association variants perturbing certain network topologies. The default STMP module comes pre-loaded with gene co-expression network topology representing gene expression microarray data from 75 normal unused human donor hearts, tissue from 49 human hearts with right- or left-ventricular hypertrophy, and 436 explanted human hearts with dilated cardiomyopathy. The general framework for weighted gene coexpression network analysis is described [22,23]. Brielfy, pair-wise Pearson’s correlation between gene expression values was calculated for every gene in the dataset for: a) samples from normal unused donor hearts, b) samples from hearts with right- or left-ventricular hypertrophy, c) samples from hearts with dilated cardiomyopathy. A soft-thresholding parameter β was chosen to satisfy scale-free topology criterion based on r2 maximization for a linear fit with slope –1 to log(k) versus log(n(k)), and the topological overlap between genes was calculated [24], generating a network adjacency based on shared network neighbors. We next used average linkage hierarchical clustering and the dynamic tree cut algorithm [25], to partition the topological overlap network into modules. Disease-specific topologies can be used to assess dynamic gene-gene interactions that are context specific.

### Disease associated variant discovery in father-mother-child trios with simplex phenotypes

Genome-wide genetic interrogation in father-mother-child trios with apparently simplex phenotypes can be a powerful tool for genetic association discovery. Classically these investigations are performed using discrete filtering to identify apparent *de novo*, compound heterozygous, and rare homozygous mutations. *De novo* mutations are typically discovered via searching for Mendelian inheritance abnormalities (MIAs) that are consistent with the segregation of phenotypes within the family. Discrete filtering is encumbered by several challenges, however. First, the true per-generation *de novo* mutation rate [26,27] is two to three orders of magnitude higher than the sequence error rate using current high throughput sequencing technology [27,28]. In addition to stochastic error modes, there are systematic error modes that relate to sequences in the human reference genome that are compressed relative to common repetitive sequences, low complexity and GC and homopolymer rich regions, and other regions of the human genome that are problematic to accurately sequence, align, or genotype. Roach, et al, developed an HMM-based classifier to identify these regions in family quartets and also to provide relative inheritance state information for WGS [27,29]. STMP utilizes a simplified hidden Markov model (HMM) classifier that bins WGS or WES data into one of three categories: “good data”, “compression”, or “MIA-rich” regions. The latter two categories represent variant data that is highly likely to contain systematic artifact and can be excluded from downstream analysis and-or interpretation. STMP writes this information in the “Filter” field of standard VCF output, allowing for soft-filtering or manual inspection of these regions. STMP uses chromosomal distance between variant markers as a prior expectation in the HMM, thereby facilitating the use of this HMM-based approach in WES, which by virtue of sequence capture is sparse outside targeted regions and dense within targeted regions.

Second, apparently simplex disease phenotypes can arise from a variety of possible underlying genetic architectures and modes of inheritance, and pre-specifying one mode can lead to a lack of sensitivity. To address this, once HMM-based regional classification has been performed, STMP will output 1) apparent *de novo* events, 2) all instances of compound heterozygosity in a gene in which at least one variant in the pair is rare according to user specific criteria, 3) rare homozygous mutations, and 4) instances of apparent hemizygosity which are candidates for loss of function, 5) rare variants in known inherited disease genes fitting an autosomal dominant inheritance model with reduced penetrance. This output can be used for manual inspection in single trio studies or as a prior expectation for gene regions fed forward into collapsing statistics if a cohort of trios is studied. In the latter case STMP leverages inheritance information to reduce the number of gene regions queried and thus the number of statistical comparisons performed between case and control cohorts.

### Drug response prediction from high throughput sequence data

Predicting drug response from WGS data requires generation of best-guess haplotypes from short-read sequence data for which haplotype phase is often not determined molecularly. STMP produces best-guess haplotype pairs from confidently genotyped SNVs and indels identified as above. To do this STMP first creates skeleton haplotype pairs using all confidently identified homozygous SNVs. The full set of complementary haplotypes is then generated using heterozygous variant calls. A perfect-match search is performed for each haplotype and its complement among described haplotypes defining the known “star” alleles associated with clinical drug response. If a perfect match is not found, the set of possible haplotype pairs is given but no star allele assignment is attempted. If more than one pair of possible star alleles is found matching possible haplotypes generated from WGS data, all possible star allele combinations are reported. STMP does not provide haplotype resolution beyond that suggested by the confidently called genotypes. That is, if a variant is not confidently called or not covered, as may be the case in exome or other targeted sequencing, haplotypes that are uniquely defined by these “tags” variants are not disambiguated from other possible star allele-defining haplotypes, and a set of possible star alleles corresponding to each reduced haplotype and its conjugate are reported. The haplotype determination is purposefully designed to only give high-confidence predictions, leaving the task of disambiguating star alleles in the setting of uncertain genotype calls or uncommon haplotypes to a human curator.

STMP also annotates and reports single variant drug response associations cataloged in the PharmGKB knowledgebase [30] at a level of evidence (for definitions, see http://www.pharmgkb.org/page/clinAnnLevels) defined by the user (**Fig 1**).

### Bioinformatic prioritization of candidate Mendelian disease risk variants

Following genotype annotation, STMP prioritizes variants by using metrics of conservation and constraint, predictions of pathogenicity, and allele frequency derived from comparisons with local and external data sources. STMP uses a prioritization scheme that at once provides a parsimonious set of candidates for manual review and a comprehensive assessment of previously reported genetic variation. The heuristic for prioritization of previously reported variants in monogenic disease genes, as well as rare and novel variants in monogenic disease genes with no previously reported phenotypic association, is described in **Fig 1.** In default mode STMP first reduces all variants to a set that occurs within 2,725 genes cataloged in ClinVar (2,716 in females due to cataloged disease associations within nine genes on the Y chromosome) [31], manually curated to exclude drug response associations and common disease susceptibility loci. Alternatively, gene sets can be provided by the user and utilized for genetic variant filtering based on the phenotypic features of the disease queried.

Previously reported variants in disease mutation catalogs include a significant number of common polymorphisms, mapping errors, legacy coordinates, and common disease susceptibility loci that are unlikely to be relevant to monogenic disease risk [32,33]. Thus, STMP prioritizes variants previously reported in the Human Gene Mutation Database (HGMD) first by expected pathogenicity and next by allele frequency. Previously reported variants cataloged in HGMD are separated into four tiers of potential pathogenicity:

- **Tier 1**: Loss of function variants (splice dinucleotide disrupting, nonsense, nonstop, and frameshift indels, large coding insertions and deletions).
- **Tier 2**: All rare variants cataloged in HGMD, regardless of functional annotation. Rarity is defined as minor allele frequency (MAF) no greater than 1% by default or according to use-defined criteria in any of the following population genetic surveys: ethnically-matched population in HapMap 2 and 3, the 1000 genomes phase 1 data[34] from an ethnically-matched super population, and global allele frequency, the 1000 genomes pilot 1 project global allele frequency, 69 publicly available genomes released by Complete Genomics, the NHLBI Grand Opportunity exome sequencing project global allele frequency, and the Exome Aggregation Consortium (http://exac.broadinstitute.org).
- **Tier 3**: All non-rare missense and non-frameshift indels.
- **Tier 4**: All variants not meeting criteria for tiers 1–3.

Variants meeting criteria for tiers 1–3 are retained for downstream manual review in the case of individual genome interpretation, and for intersection with inheritance state information in the case of disease-targeted analyses. As the expected allele frequency of potentially pathogenic variants is likely to vary greatly with disease prevalence, penetrance, and mode of inheritance, allele frequency filters are not used for tiers one and three, thereby allowing for prioritization of functional alleles with previously described disease associations that would not otherwise pass strict allele frequency filters, for instance the deltaF508 allele in *CFTR* or the Factor V Leiden allele.

Rare and private variation, as a result of recent population expansion and purifying selection, continues to constitute a significant proportion of human genetic variation, even in large population surveys. Some of these rare and private alleles will have monogenic disease risk and carrier status relevance, and therefore STMP also prioritizes select previously unreported, but potentially pathogenic, rare and novel variants in monogenic disease genes, incorporating consensus evidence for evolutionary constraint/conservation and pathogenicity prediction. These computational methods for scoring genetic variants have, in isolation, modest classification accuracy and inter-algorithm concordance [35,36]. Approaches to rating the potential pathogenicity of variants based on consensus of commonly used prediction algorithms have been shown to have superior calibration and discriminative accuracy when compared with individual predictions [36]. STMP imposes a prior expectation that pathogenic alleles are more likely to occur in genes in which previously reported variants have produced Mendelian disease phenotypes, but also archives and categorizes all other variants for review and potential reclassification as genetic knowledge expands, or for intersection with inheritance state data. Rare (defined as above) and novel variants with no previously described phenotype association in monogenic disease genes are prioritized into four tiers of potential pathogenicity:

- **Tier 1**: Rare loss of function variants, defined as above.
- **Tier 2**: Rare missense variants affecting nucleotides with consensus evidence by both algorithms considered for evolutionary conservation/constraint according to the following values cataloged in the database of nonsynonymous functional predictions [35]: GERP++ score > 2; PhyloPnew >0.95 and rare non-frameshift indels.
- **Tier 3**: Rare missense variants with predicted pathogenicity by three or more algorithms according to the following definitions as cataloged in the database of nonsynoymous functional predictions [35]: SIFT, “Damaging”; LRT, “Deleterious”; PolyPhen2, “Probably damaging” or “Possibly damaging”; Mutation taster, “Disease causing automatic” or “disease causing”.
- **Tier 4**: All rare variants not meeting criteria for tiers 1–3.

As a final filter for both previously reported variants in monogenic disease genes and previously unreported, rare variants in monogenic disease genes, STMP uses catalogs of local allele frequency and genotype information to exclude variants that are observed more frequently than would be expected in the local sequencing environment. This filter serves to identify and exclude variants whose previously unappreciated high allele frequency is a result of false negatives in population genetic surveys or systematic false positives specific to local sequence variant discovery pipelines. Similar filters have proven effective in excluding such systematic artifacts in other contexts [37].

### Inputs, implementation and parallelization

STMP accepts as required input a vcf file prepared by Illumina Isaac, GATK, the Complete Genomics variant discovery pipeline, or Real Time Genomics. Optional inputs include 1) binary alignment map file, required for targeted genotype calling and annotation of known inherited disease risk alleles and pharmacogenomic annotation, 2) genome feature format file describing structural variant events, 3) local site frequency spectrum for filtering of inherited disease risk candidates. In trio mode STMP requires jointly called genotypes and sample identifiers for father, mother, and child. Initial annotation of genetic variants for gene, functional genomic, and clinical phenotypes is performed using python/perl and parallelized using the “multiprocessing” module in python. Processing time for the annotation pipeline scales roughly as 1/n, where n is the number of processors allocated to the task. STMP is implemented in cython/python/shell and also parallelized using the “multiprocessing” modules in python. To demonstrate the utility of the STMP tool, we applied STMP in trio mode to a trio with congenital neonatal arrhythmia, and also to twelve unrelated adult participants (median age 53, 6 female, 7 of East Asian ancestry) recruited from primary care clinics at Stanford University Medical center. The study was approved by the Stanford University Institutional Review Board and all patients gave informed written consent, or, in the case of minors, assent (Stanford Institutional Review Board GAP approval number 4237).

### Functional regulatory analysis of common variants discovered by STMP

To further explore a novel gene locus implicated in the congenital neonatal arrhythmia trio, we performed *in vitro* characterization of a putative promoter variant found in *trans* with a novel protein truncating mutation in *ATP2B4* using previously described approaches[38]. The noncoding variant in the 5’ un-translated region of *ATP2B4*, rs4600103, was first investigated *in silico* using 1000 Genomes haplotype data and ENCODE regulatory datasets for chromatin accessibility and histone modifications. Predicted transcription factor binding sites (TFBS) altered by rs4600103 and the linked variant (r^2^~0.87), rs4951276 (also present in the patient), were determined using TRANSFAC, JASPAR, and MatInspector positional weight matrix (PWM) databases. Sequences surrounding each allele were scanned for vertebrate TFBS with a 0.9 minimum similarity score cutoff. Identified putative enhancer elements for each SNP were used to generate concatenated oligonucleotides for rs4600103-G/A and rs4951276-T/A, which were annealed at 95 C for 5 minutes and allowed to cool to room temperature. Resulting double-stranded DNA fragments were subcloned into the multiple cloning site (MCS) of the pLuc-MCS vector (Agilent), and constructs were confirmed by Sanger sequencing. Luciferase reporter constructs containing respective major and minor alleles of rs4600103 and rs4951276 were transfected, along with renilla luciferase, into HEK 293 and H9c2 cells using Lipofectamine 2000 (Life Technologies), according to the manufacturer’s instructions. Growth media was changed after 5 hours and dual-luciferase activities measured after 24 hours using a SpectraMax luminometer (Molecular Devices). Firefly luciferase activities were normalized to renilla luciferase and expressed as the fold change of the empty vector control.

## Results

### STMP identifies a candidate gene for neonatal ventricular arrhythmia in a father-mother-child trio

**Fig 2:**
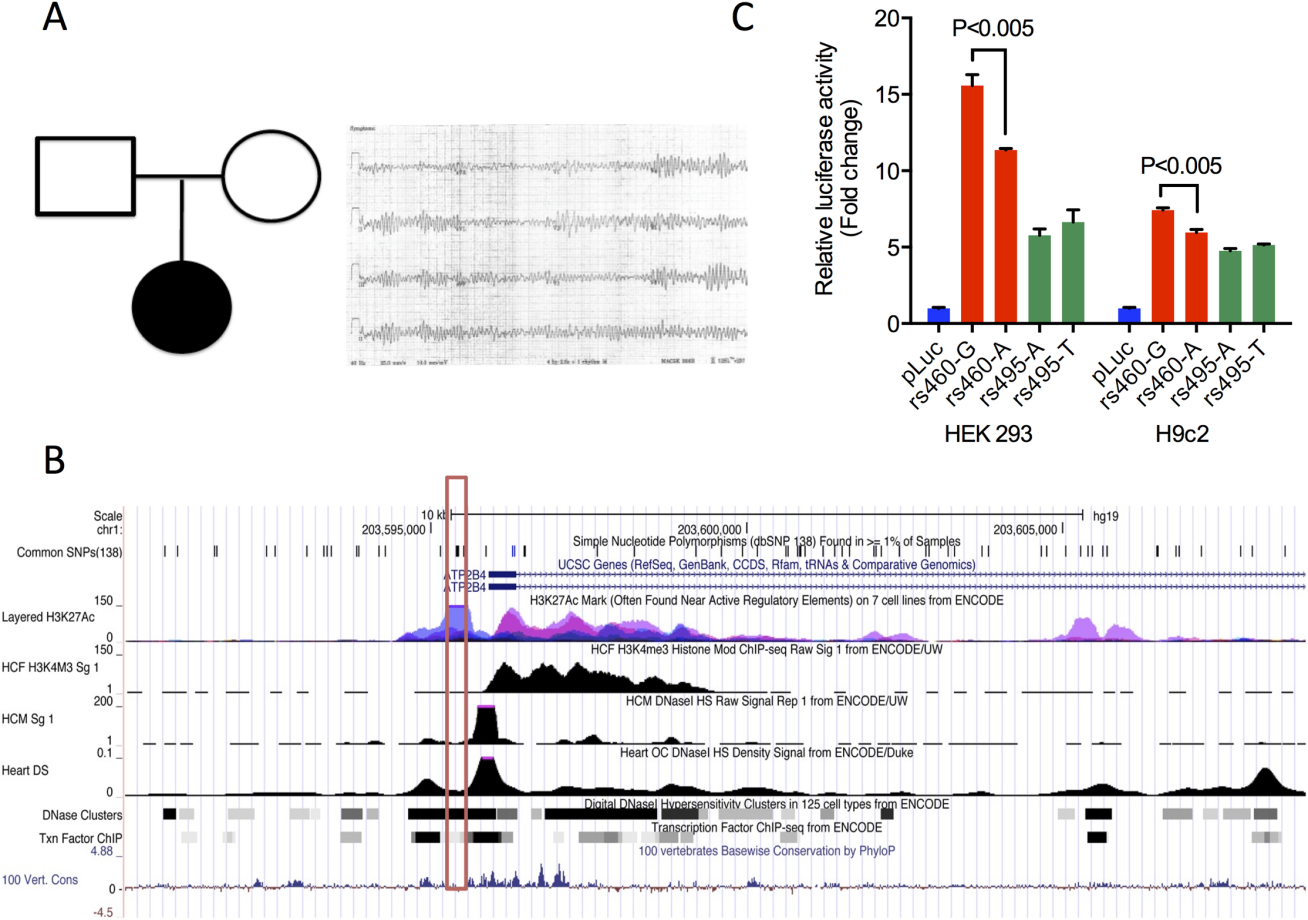
STMP identifies a likely functional regulatory variant in a novel candidate disease gene for neonatal ventricular arrhythmia. A) Pedigree (left) and representative neonatal ECG from a proband with ventricular fibrillation (right). B) UCSC Genome Browser screenshot showing ENCODE regulatory tracks surrounding a novel variant in the 5’ UTR, rs4600103 (red box), found in *cis* with a nonsense variant in *ATP2B4*, as well as linked variant (r^2^ = 0.87) rs4951276 (green box). Tracks for chromatin accessibility, including DNaseI hypersensitivity, and promoter histone modification (H3K4M3) ChIP-seq data are shown for human cardiac myocytes (HCM), human cardiac fibroblasts (HCF) and heart tissue. DNaseI hypersensitivity clusters, transcription factor ChIP-seq and active histone modification (H3K27Ac) ChIP-seq data are shown for multiple ENCODE cell lines. C) Functional validation of common variants using allele-specific reporter assays. Common variants at *ATP2B4*, rs4600103 and rs4951276 were evaluated in luciferase reporter assays in HEK293 and H9c2. Values are expressed as relative fold change versus empty vector (pLuc) and represent mean ± SEM of triplicates from three independent experiments.

The trio format is a common familial arrangement in sequencing studies undertaken to uncover the genetic basis for a known disease or to assist in disease diagnosis [39,40]. In many such trios, the offspring is the only clearly affected subject (“simplex” trios), proposing several possible modes of phenotypic expression, including recessive or codominant inheritance, autosomal dominant inheritance with reduced penetrance, and a *de novo* mutation in the proband. STMP functionally annotates and prioritizes such alleles, including all possible instances of compound heterozygosity and important noncoding variants. To demonstrate the utility of STMP for discovery of disease-associated genetic variants in individuals with manifest disease in this format, we used STMP in “trio” mode to investigate whole genome sequence data from a father-mother-child trio with neonatal ventricular arrhythmia. In this trio the offspring was affected by neonatal polymorphic ventricular arrhythmia preceded by ST segment elevation (**Fig 2A.**). Clinical genetic testing of inherited arrhythmia genes, including deletion-duplication testing, did not uncover disease-causing mutations. STMP identified 25 candidate variants. Among the candidate compound heterozygous variants, a novel nonsense mutation and a common 5’ UTR variant were found *in trans* in *ATP2B4;* the latter variant, rs4600103, was found in an accessible and active chromatin region as determined by ENCODE derived DNAse hypersensitive regions and enrichment for promoter histone modifications (H3K4me3) in human cardiac fibroblasts (HCF) and cardiac myocytes (HCM) and active histone modifications (H3K27ac) in human lung fibroblasts (**Fig 2B**). *ATP2B4* encodes a plasma membrane calcium ATPase that mediates neuronal nitric oxide signaling in cardiac myocytes and directly interacts with a gene, *SNTA1*, that has been implicated in hereditary ventricular arrhythmia and sudden, presumed arrhythmic infant death [41–43]. Using TRANSFAC and JASPAR transcription factor binding databases, we identified altered motifs for ELK1 and NFκB transcription factor binding sites (TFBS) proximal to the SNP. Using 1000 Genomes data we identified another common variant, rs4951276 (MAF 0.35 ASN, 0.09 EUR) in high linkage disequilibrium (r^2^ = 0.87) with rs4600103, which may explain some of the regulatory effects. This intronic variant resides in a putative enhancer element, containing the active chromatin mark, H3K27ac, and is predicted to disrupt TFBS motifs for FOXP1. To interrogate the potential impact of these variants in regulating *ATP2B4* expression, we cloned the predicted regulatory elements surrounding each allele into a luciferase reporter construct driven by a minimal promoter, and measured relative transcriptional activity in both HEK 293 and the neonatal rat cardiomyocyte cell line, H9c2. Interestingly, the minor A allele at rs4600103 was shown to have reduced transcriptional activity compared to the major G allele, whereas both alleles at rs4951276 had similar reporter activities (**Fig 2C**). These results indicate that rs4600103 may be a functional variant identified at *ATP2B4* through altering a putative cis-regulatory element. It remains unclear whether this variant, in combination with the truncating variant in trans, is disease associated. It may be that an as yet unidentified factor such as a trans-acting regulatory element, structural variant on the other allele, or environmental- or gender-specific modifier of the phenotype is at play. This uncertainty highlights one of the challenges inherent to identifying a single likely pathogenic allele in a recessive disease gene.

### Drug response predictions from WGS data

When run on WGS data from a single proband, STMP provides, for the first time, pharmacogenomic haplotype assignment and annotation of clinically associated pharmacogenomics alleles, including those that are defined on a single variant or haplotype bases. To demonstrate the utility of STMP in this context, we assessed the concordance between star allele assignments generated by STMP for five genes with associated Clinical Pharmacogenomics Implementation Consortium (CPIC) guidelines for drug dosing and administration (*CYP2C9*, *CYP2C19*, *CYP2D6*, *VKORC1*, and *SLCO1B1*). Haplotype call concordance between STMP and blind manual haplotype determination demonstrated that in all twelve individuals, the star allele pair assigned by human rates was found in the set of possible star alleles reported by STMP for all five genes. As described [44], STMP provided 1–3 recommendations per subject for change in drug dose or administration, and 3–10 additional high-confidence genetic drug response predictions from WGS data.

### Identification of putative Mendelian disease risk alleles from WGS data

When applied to WGS data from single probands, STMP provides rich functional annotation and prioritization of potential Mendelian disease risk alleles, including novel variants, structural variants, and important regulatory variants. STMP allows for genome-wide search for such genetic variants, or can be restricted to specific gene sets if a targeted diagnostic question is pursued. To demonstrate the utility of STMP to discover such variants genome-wide, we applied STMP to Illumina WGS sequence data (median haploid read depth 51x, 101bp x 2 paired end reads, generated on the HiSeq 2000) from twelve unrelated adult participants (median age 53, 6 female, 7 of East Asian ancestry) recruited from primary care clinics at Stanford University Medical center. Methods for identification of single nucleotide variants, indels, and structural variants are described in Dewey, et al (2014) [44]. On a six-core Intel Xeon X5670 processor running 64-bit linux with 128 GB of RAM and utilizing five concurrent threads, stanovar performed comprehensive annotation of standard.vcf and.gff format variant files in a median of 96 (range 90–102) minutes per genome. STMP performed prioritization of Mendelian disease risk candidates and identification of genotypes and haplotypes affecting drug response in < 5 minutes per participant. The median total processing time, including targeted genotype calling of SNVs and indels with clinical associations and filtering based on local site frequency spectra, was 122 minutes (range 116–127 minutes). We used allele frequency filters of <1% in general population surveys and <25% in our local cohort; higher allele-frequency cutoffs for local sequence data may be appropriate in populations enriched for Mendelian phenotypes and associated variant alleles. Higher allele-frequency cutoffs for local sequence data may be appropriate in populations enriched for Mendelian phenotypes and associated variant alleles. Manual curation uncovered several well-established disease causing mutations in this cohort without apparent Mendelian disease, including a 19-bp insertion-deletion variant in *BRCA1* that has been previously implicated in hereditary breast and ovarian cancer, prompting prophylactic surgery [44].

Variants discovered in each participant, prior to and after allele frequency filtering, are presented in **Table 1**. Further filtering of variants occurring at high allele frequencies in the cohort was particularly effective at reducing the number of previously reported Mendelian disease risk candidates and the number of apparently rare (according to external allele frequency information) loss-of-function variants in Mendelian disease genes. This suggests that even a small number of local “control” genomes can substantially reduce the number of potential false positives resulting from systematic sequencing artifact related to the local peculiarities of sequencing and analysis or previously unappreciated common variation.

**Table 1:** Summary of STMP tiers 1–3 in whole genome sequence data from twelve unrelated adults recruited from primary care clinics.

Variant classification	Definition	Median (range)–all variants	Median (range)–filtered by cohort allele frequency	% private	Variant alleles present in all participants, n	Variant alleles present in all East Asians, n
Genetic variants with previously reported disease associations						
Tier 1	Loss of function	8.5 (6–14)	3.5 (2–9)	15 (0–36)	0	0
Tier 2	Rare non-LOF variants	147 (124–164)	14 (5–76)	3 (2–36)	1	8
Tier 3	Common missense and nonframeshift indel variants	148.5 (133–154)	29 (26–68)	6 (4–22)	2	13
Rare* variants in Mendelian disease genes with no reported disease association	—	62,453 (59,813–66,207)				
Tier 1	Loss of function	13 (11–19)	6 (3–13)	23 (7–47)	2	2
Tier 2	Missense/nonframeshift+ conserved	53.5 (48–59)	50.5 (42–55)	66 (54–73)	1	1
Tier 3	Missense/nonframeshift+ predicted pathogenic	5 (4–8)	5 (4–8)	100 (60 - 100)	0	0

*As defined by allele frequency < 0.01.

Abbreviations: LOF, loss of function.

## Discussion

Here we describe a series of methods for annotation of high-throughput sequence data for individual genetic risk prediction and prediction of drug response. This collection of tools is flexible, customizable, and allows for dynamic interaction between variant annotation and association efforts. It is applicable to variant data from whole genome, exome, and targeted re-sequencing. We further demonstrate that application of these methods to whole genome sequence data in apparently unrelated individuals yields a parsimonious set of variants for manual review (~100) that may have implications for Mendelian disease risk and drug response, and in one case uncover a clearly clinically actionable disease-causing mutation in a pre-symptomatic individual. Application to a father-mother-child trios uncovered a novel candidate disease gene in neonatal ventricular arrhythmia. While several methods exist for predicting pathogenicity of sequence variants, and other methods exist for annotating variants with respect to described disease associations, there is not yet a unified framework that integrates the bulk of human disease-genotype associations and computational predictions. The set of methods we described here is developed to do just that. Furthermore, in contrast to existing tools that perform limited annotation of SNVs and indels, our integrated pipeline provides a framework for interpreting structural variants and variants disrupting important noncoding regions of genes associated with disease phenotypes. This set of methods leads naturally into manual curation of discovered variants for research efforts utilizing disease phenotype and drug response information.

Sequence to medical phenotypes (STMP) is an open source, parallelized pipeline for clinical interpretation of WGS and WES data generated in a research setting. It is highly amenable to parallel processing architecture, produces parsimonious variant sets for manual review, and interrogates both Mendelian disease risk and genetic drug response. We hope that the methods presented here will help catalyze future clinical research using WGS.

STMP is open source and will be available at http://ashleylab.stanford.edu/tools/stmp.html.

## Supporting Information

S1 Table Annotations for single nucleotide and insertion/deletion variants. (XLSX)

S2 Table Annotations for structural variants. (XLSX)
